# Health Symptoms Related to Polycyclic Aromatic Hydrocarbon (PAH) Exposure in Chiang Mai, Thailand: Associations with Biomarkers of Exposure and Oxidative Stress

**DOI:** 10.3390/toxics13090796

**Published:** 2025-09-18

**Authors:** Xianfeng Cao, Sumed Yadoung, Phannika Tongchai, Anurak Wongta, Kanokwan Kulprachakarn, Peerapong Jeeno, Pichamon Yana, Udomsap Jaitham, Wenting Li, Kai Zhou, Xiao Zhang, Jianmei Gong, Natthapol Kosashunhanan, Surat Hongsibsong

**Affiliations:** 1 School of Health Sciences Research, Research Institute for Health Sciences, Chiang Mai University, Chiang Mai 50200, Thailand; xianfeng_cao@cmu.ac.th (X.C.); phannika_tongchai@cmu.ac.th (P.T.); anurak.wongta@cmu.ac.th (A.W.); kanokwan.kul@cmu.ac.th (K.K.); peerapong_jeen@cmu.ac.th (P.J.); udomsap_j@cmu.ac.th (U.J.); wenting_l@cmu.ac.th (W.L.); natthapol.ko@cmu.ac.th (N.K.); 2 Environmental, Occupational Health Sciences and NCD Research Group, Research Institute for Health Sciences, Chiang Mai University, Chiang Mai 50200, Thailand; sumed.yadoung@cmu.ac.th (S.Y.); pichamon.y@cmu.ac.th (P.Y.); 3 Office of Research Administration, Chiang Mai University, Chiang Mai 50200, Thailand; 4 Jiujiang Key Laboratory of Food Processing and Safety, School of Pharmacy and Life Sciences, Jiujiang University, Jiujiang 332005, China; zhoukaitougao@163.com; 5 School of Mechanical and Intelligent Manufacturing, Jiujiang University, Jiujiang 332005, China; zxjju2019@163.com; 6 School of Nursing, Liaoning University of Traditional Chinese Medicine, Shenyang 110847, China; damei511@163.com

**Keywords:** polycyclic aromatic hydrocarbons, oxidative stress, respiratory symptoms, club cell protein 16 (CC16), 8-iso-prostaglandin F_2_α (8-iso-PGF_2_α)

## Abstract

Northern Thailand experiences seasonal surges in PM_2_._5_ pollution, posing significant respiratory health risks. This cross-sectional study aimed to evaluate associations between PAHs exposure and early health biomarkers. In April 2024, 127 rural residents in Chiang Mai were recruited during a high-exposure period (mean monthly PM_2.5_ = 41.7 μg/m^3^). Participants reporting eye irritation and pneumonia showed significantly higher 8-iso-PGF_2_α levels (*p* = 0.010 and 0.012, respectively). Smokers exhibited elevated CC16 levels (130.0 ± 65.3 ng/mL) compared to non-smokers (96.3 ± 39.9 ng/mL, *p* < 0.05). CC16 was also significantly associated with self-reported symptoms, including fatigue, poor sleep quality, and activity limitation. For example, participants who reported difficulty performing daily activities (i.e., disagreed with the statement “I can do things at home without any restrictions”) had significantly higher CC16 levels (108 ± 47 ng/mL) than those without such limitations (74 ± 35 ng/mL; *p* < 0.001). A weak but significant positive correlation was observed between respiratory rate and CC16 (R^2^ = 0.334, *p* = 0.001). Interestingly, serum 8-iso-PGF_2_α was inversely associated with diabetes (OR = 0.965; 95% CI: 0.935–0.997; *p* = 0.033), potentially indicating a compensatory or phenotype-specific oxidative stress response. In addition, CC16 levels were positively associated with diabetes (*p* = 0.022), suggesting altered epithelial responses in individuals with metabolic disease. CC16 and 8-iso-PGF_2_α demonstrated significant associations with respiratory symptoms and metabolic status, suggesting their potential as early indicators for environmental health surveillance in haze-affected populations.

## 1. Introduction

Air pollution, particularly Particulate Matter (PM) and Polycyclic Aromatic Hydrocarbons (PAHs), has emerged as a major environmental health threat in Southeast Asia, including northern Thailand [1,2]. Chiang Mai, in particular, experiences severe seasonal air pollution episodes, with PM_2.5_ levels frequently surpassing national and international guidelines [3]. Prolonged exposure to air pollutants has been linked to a range of respiratory and cardiovascular diseases, disproportionately affecting vulnerable populations such as the elderly and those with pre-existing chronic conditions [4].

To monitor early health impacts of environmental exposure, biomarkers offer a promising strategy for detecting subtle physiological changes before the onset of overt disease. Urinary 1-hydroxypyrene (1-OHP), a major metabolite of pyrene, is widely recognized as a sensitive biomarker for PAH exposure and has been associated with particulate matter exposure and systemic oxidative-inflammatory responses in both children and adults [5]. Serum Club Cell Protein 16 (CC16), secreted by non-ciliated bronchiolar epithelial cells, serves as an indicator of lung epithelial integrity and has been shown to decline in individuals chronically exposed to ambient and traffic-related air pollution [6]. Additionally, 8-iso-prostaglandin F_2_α (8-iso-PGF_2_α), a lipid peroxidation product, has emerged as a reliable oxidative stress marker in respiratory and cardiovascular conditions [7].

Together, these biomarkers reflect complementary pathophysiological processes—PAH metabolism (1-OHP), epithelial damage (CC16), and oxidative injury (8-iso-PGF_2_α)—providing a multi-dimensional perspective on pollution-related health effects. However, most existing studies have focused on clinical populations or controlled exposure settings. Few investigations have simultaneously evaluated these biomarkers in relation to self-reported respiratory symptoms, fatigue, or functional limitations in community-based adult populations under real-world pollution conditions.

This study aims to fill this gap by examining the associations between urinary 1-OHP, serum CC16, and 8-iso-PGF_2_α levels and self-reported symptoms and health perceptions among rural residents in Chiang Mai during a high PM_2.5_ period. By integrating biomarker assessments with structured questionnaire responses, we seek to evaluate the potential of these biomarkers as early warning indicators for environmental health surveillance in high-risk regions.

## 2. Materials and Methods

### 2.1. Study Design and Participants

This cross-sectional study was conducted in April 2024, during the high PM_2.5_ season in Chiang Mai Province, northern Thailand. Data collection took place in Thung Sathoek Subdistrict, a rural community within San Pa Tong District, selected due to its historically high levels of ambient PM_2.5_, as reported by the regional air quality monitoring network. A total of 127 adult participants were enrolled through convenience sampling, supported by local village health volunteers. Inclusion criteria comprised adults aged 18 years or older who had resided in the area for at least six months. Individuals with active respiratory infections, known malignancies, or current corticosteroid use were excluded. A priori sample size estimation using G*Power (Version 3.1) (for correlation analysis, effect size = 0.5, α = 0.05, power = 0.80) indicated that a minimum of 85 participants would be required to detect medium effect sizes, confirming the adequacy of the study’s sample.

### 2.2. Ethical Approval and Consent

The study protocol was reviewed and approved by the Ethics Committee of the Faculty of Associated Medical Sciences, Chiang Mai University (Protocol No. AMSEC-66EX-062 8/60, dated 3 November 2023). Participants provided written informed consent after being thoroughly informed of the study objectives, procedures, data confidentiality, and their rights to withdraw without consequences, in accordance with the Declaration of Helsinki.

### 2.3. Questionnaire and Data Collection

Data were collected through structured, face-to-face interviews using a validated Thai-language questionnaire adapted from the Health Impact Assessment Division, Ministry of Public Health (2021) [8]. The questionnaire demonstrated acceptable internal consistency (Cronbach’s α > 0.75) and covered three domains: participants information (including gender, age, smoking history, Body Mass Index), self-reported symptoms and perceived health risks related to PAHs. Anthropometric data (height and weight) were measured onsite using standardized equipment; age was confirmed via national ID or personal recall. Symptom responses (e.g., “I sleep well”) were rated on a binary scale (0 = disagree, 1 = agree), and were analyzed as categorical variables.

### 2.4. PM_2.5_ Exposure Measurement

Daily ambient PM_2.5_ concentrations were reported by the Northern Thailand Air Quality Health Index (NTAQHI) platform, managed by the Pollution Control Department (PCD), Ministry of Natural Resources and Environment. Monthly average concentrations were calculated based on daily 24 h mean values recorded at the Tung Satok monitoring station in Chiang Mai Province. These monthly averages were used to characterize seasonal air pollution trends and contextualize the timing of participant recruitment and sampling. However, these data reflect ambient background levels and do not account for individual-level exposure variations such as time spent indoors or use of protective equipment.

### 2.5. Biomarker Collection and Laboratory Analysis

#### 2.5.1. Urine Sampling and 1-OHP Measurement

Participants provided first-morning urine samples using sterile polyethylene containers. Samples were transported on ice to Research Institute for Health Sciences, Chiang Mai University, and stored at −80 °C until analysis. Urinary 1-OHP was measured using high-performance liquid chromatography (HPLC-FLD; Agilent 1260 Infinity II, Santa Clara, CA, USA) with fluorescence detection [9]. Creatinine concentration was quantified using the Jaffe method, and 1-OHP values were reported as μmol/mol creatinine. Quality control included certified reference materials, blank samples, duplicate runs (10% of samples), and calibration curves with R^2^ > 0.99.

#### 2.5.2. Serum CC16 and 8-iso-PGF_2_α

A 3 mL venous blood sample was drawn from each participant under aseptic conditions by trained phlebotomists. After centrifugation (4000× *g*, 15 min, 4 °C), serum was aliquoted and stored at −80 °C. CC16 (Clara cell secretory protein) levels were quantified using a commercial ELISA kit (Guangzhou AORUIDA Biotechnology Co., Ltd., Guangzhou, China), with sensitivity < 1 ng/mL and inter-assay CV < 10%. 8-iso-PGF_2_α, a lipid peroxidation marker, was measured in urine using a competitive ELISA kit (Guangzhou AORUIDA Biotechnology Co., Ltd., Guangzhou, China). Serum concentrations of CC16 and 8-iso-PGF_2_α were expressed in ng/mL without creatinine normalization. Each assay followed the manufacturer’s instructions, with duplicate samples analyzed to ensure reliability.

### 2.6. Statistical Analysis

All statistical analyses were conducted using SPSS version 27 (IBM Corp., Armonk, NY, USA). Descriptive statistics were reported as mean ± SD for continuous variables and frequencies (%) for categorical variables. Data normality was assessed using the Shapiro–Wilk test. For bivariate analysis: Spearman rank correlation was used for non-normally distributed or ordinal data (e.g., CC16 and respiratory rate, questionnaire scores). To provide a more comprehensive evaluation, multivariate regression models were employed. Specifically, binary logistic regression was used to assess associations between dichotomized self-reported symptom questionnaire items (dependent variables) and biomarker levels (independent variables), adjusting for potential confounders including age, sex, and smoking status. In addition, multivariate linear regression models were constructed to evaluate the association between health biomarkers (e.g., CC16, 8-iso-PGF_2_α) and clinical or behavioral predictors, adjusting for potential confounders including age, gender, smoking status, use of masks and air purifiers, as well as other biomarkers (1-OHP, CC16, and 8-iso-PGF_2_α as appropriate) [10]. Final adjusted models were selected based on biological plausibility and statistical significance in univariate analysis (*p* < 0.20), and the best-fitting model was confirmed using the Akaike information criterion (AIC) [11]. A two-sided *p*-value < 0.05 was considered statistically significant. Correlation heatmaps were generated using GraphPad Prism (version 10; GraphPad Software, San Diego, CA, USA) to visualize biomarker-symptom associations.

## 3. Results

### 3.1. Demographic and Exposure Characteristics of Participants

A total of 127 participants were included, with a mean age of 59.7 ± 7.9 years and a predominance of males (74%) in Table 1. Only 15.7% of participants reported a smoking history. The average BMI was 24.3 ± 8.1 kg/m^2^. Biomarker analysis showed a mean urinary 1-hydroxypyrene (1-OHP) level of 0.88 ± 1.26 μmol/mol creatinine, indicating varied PAH exposure. Serum CC16 averaged 101 ± 48 ng/mL, and 8-iso-prostaglandin F2α (8-iso-PGF2α) averaged 52.5 ± 20.6 ng/mL, reflecting potential differences in airway integrity and oxidative stress.

### 3.2. Associations Between Participant Characteristics and Biomarker Levels

The comparisons of biomarker levels stratified by demographic and lifestyle characteristics revealed that the mean serum CC16 level was significantly higher in participants with a history of smoking compared to non-smokers (130.09 ± 65.33 ng/mL vs. 96.37 ± 39.96 ng/mL, *p* < 0.05) in Table 2. No statistically significant differences were observed in the levels of CC16, 8-iso-PGF2α, or 1-OHP across categories of sex, age, BMI, or alcohol consumption (*p* > 0.05 for all comparisons). Although smokers showed numerically higher levels of serum 8-iso-PGF2α and urinary 1-OHP, these differences did not reach statistical significance.

### 3.3. Monthly Variation in PM_2.5_ Concentrations (September 2023–September 2024)

**Figure 1:**
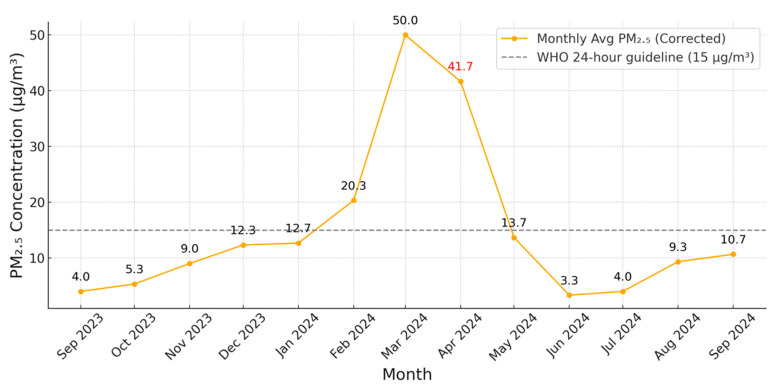
Monthly Average PM_2.5_ Concentration in Chiang Mai (September 2023–September 2024).

From September 2023 to September 2024, the monthly average PM_2.5_ concentration in Chiang Mai exhibited a clear seasonal trend in Figure 1. Levels began to rise in November 2023, peaked in March 2024 (approximately 50 μg/m^3^), and declined sharply thereafter. In April 2024, when sample collection was conducted, the monthly mean concentration was still 41.7 µg/m^3^. Sample collection for biomarker and symptom assessment took place in April 2024, shortly after the seasonal PM_2.5_ peak, allowing for evaluation of post-exposure health effects.

### 3.4. Associations Between Biomarker Levels and Self-Reported Symptoms

After adjusting for potential confounders (age, sex, and smoking status), several significant associations between biomarker levels (urinary 1-OHP, serum CC16, and 8-iso-PGF2α) were observed. Notably, lower CC16 levels were significantly associated with an increased likelihood of eye irritation (*p* = 0.002), skin irritation (*p* = 0.047), pneumonia (*p* = 0.002), and loss of consciousness (*p* = 0.043). In addition, elevated 8-iso-PGF2α levels were significantly associated with eye irritation (*p* = 0.010) and pneumonia (*p* = 0.012). Regarding chronic disease, higher CC16 levels were positively associated with diabetes (*p* = 0.022), whereas higher 8-iso-PGF2α was negatively associated with diabetes (*p* = 0.033). No significant associations were observed between 1-OHP and any symptom (Table 3).

### 3.5. Adjusted Associations Between Self-Reported Respiratory Symptoms and Biomarker Levels (CC16, 8-iso-PGF_2_α, and 1-OHP)

Multivariate binary logistic regression models were conducted to assess associations between biomarker levels across dichotomized responses to eight self-reported health and function questionnaire items, adjusting for age, sex, and smoking status. Significant associations were found between CC16 concentrations and several questions. Specifically, CC16 levels were significantly higher among participants who disagreed with the following statements: “I have never had a cough” (*p* = 0.003), “When I walk up a hill or up a flight of stairs, I can still breathe easily” (*p* = 0.001), “I can do things at home without any restrictions” (*p* = 0.001), “I sleep very well” (*p* = 0.001), and “I feel very energetic” (*p* = 0.001). These results indicate that better self-reported respiratory health was associated with higher CC16 levels, potentially reflecting preserved epithelial integrity. For 8-iso-PGF_2_α, no statistically significant associations were observed across the eight questionnaire items. Similarly, no significant associations were found between urinary 1-OHP levels and any of the questionnaire responses (all *p* > 0.05), suggesting that internal PAH exposure was not clearly linked to subjective symptom perception (Table 4).

### 3.6. Association Between Respiratory Rate and CC16 After Adjustment for Covariates

**Figure 2:**
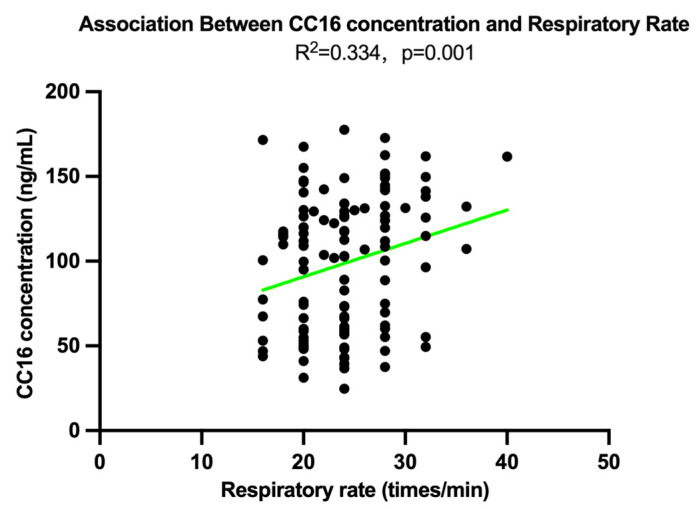
Adjusted linear association between respiratory rate (measured as the number of breaths per minute during resting conditions) and serum CC16 concentration after outlier removal. The association was examined using a multivariate linear regression model adjusting for age, sex, and smoking status (R^2^ = 0.334, *p* = 0.001).

Multivariate linear regression was conducted to examine the association between respiratory rate (breaths per minute at rest) and CC16 concentration after removing one extreme outlier. After adjusting for age, sex, and smoking status, the analysis revealed a moderate positive association between respiratory rate and CC16 concentration (R^2^ = 0.334, *p* = 0.001), as shown in Figure 2.

## 4. Discussion

The mean ambient PM_2.5_ concentration during our sampling period in Chiang Mai reached approximately 40 μg/m^3^ in March 2024. This reflects the persistent seasonal haze pattern in Northern Thailand, commonly driven by biomass burning and agricultural residue combustion during the dry season [12]. Notably, the observed level substantially exceeds both the WHO 2021 guideline of 15 μg/m^3^ for 24 h average PM_2.5_ [13] and the Thai national standard of 37.5 μg/m^3^ [13,14]. Similar seasonal surges in PM_2.5_ have been repeatedly documented in Chiang Mai and surrounding provinces [8], and such exposures have been linked to adverse respiratory outcomes, including oxidative stress, airway inflammation, and epithelial injury [15,16].

In our study, serum CC16 concentrations were significantly influenced by smoking status, with smokers exhibiting notably higher mean levels (130.09 ± 65.33 ng/mL) compared to non-smokers (96.37 ± 39.96 ng/mL, *p* < 0.05). In contrast, no significant differences in CC16 were observed between males and females or between older and younger participants. This finding contrasts with the established notion that smoking leads to reduced CC16 levels, as supported by multiple studies indicating that cigarette smoke exposure causes club cell depletion and epithelial damage, thereby diminishing CC16 secretion [17,18,19]. However, the elevated CC16 levels observed among smokers in our study may reflect a context-specific compensatory response, potentially induced by concurrent haze exposure during the biomass burning season in Northern Thailand. In such scenarios, club cells temporarily increased CC16 secretion in response to airway oxidative stress or inflammation, as suggested by recent studies on biomass smoke exposure [20]. Beyond smoking status, CC16 levels also demonstrated significant associations with a range of self-reported respiratory and general health symptoms. Notably, individuals reporting fatigue, poor sleep quality, limited daily activities, or difficulty climbing stairs exhibited markedly lower CC16 concentrations. For instance, participants with severe activity limitation had a mean CC16 level of 110.63 ± 49.83 ng/mL, which was significantly higher than that of those without such limitation (58.68 ± 24.10 ng/mL, *p* = 0.001). Similarly, CC16 was lower among participants with symptoms such as eye irritation, pneumonia, and lack of energy, suggesting its potential as an indicator of subjective discomfort during haze periods. These findings resonate with broader biomonitoring evidence showing that urinary CC16 rapidly rises after short-term PM exposure in various age groups [21]. In occupational settings, Neumann et al. (2024) reported acute rises in serum CC16 among miners after exposure to diesel and fire gases [22]. In this study, however, participants with symptoms exhibited lower CC16 concentrations, which may reflect depletion of club cells or impaired secretory capacity under sustained or repeated haze exposure. In our study, CC16 levels showed a weak but statistically significant positive correlation with respiratory rate (R^2^ = 0.334, *p* = 0.001). As the study showed, CC16 levels were positively associated with small airway function (e.g., FEF_25–75_) in a longitudinal birth cohort [23]. This relationship may suggest that increased respiratory effort, potentially linked to subclinical airway inflammation, is associated with enhanced epithelial permeability and subsequent CC16 leakage into the circulation, rather than actual upregulation in protein synthesis. The leakage mechanism has been observed in controlled exposure studies, where serum CC16 rose rapidly after pollutant inhalation, without corresponding increases in gene expression or secretion capacity [21].

In this study, serum 8-iso-prostaglandin F2α (8-iso-PGF2α), a validated marker of oxidative stress, was significantly elevated among participants reporting eye irritation and pneumonia symptoms (*p* = 0.010 and *p* = 0.012, respectively). The concurrent rise in 8-iso-PGF_2_α during high-PM_2.5_ periods reflects heightened oxidative stress, in line with prior evidence that particulate pollution triggers lipid peroxidation. For instance, traffic-related PM exposure has been linked to increased exhaled 8-isoprostane concentrations in exposed adults [24], and controlled exposure studies show PM_2.5_ can induce oxidative damage and inflammation in respiratory tissues [25]. The linkage between F2α elevations and eye irritation supports mechanistic evidence for oxidative stress-driven ocular surface damage. Airborne pollutants, especially fine particulate matter (PM_2.5_) and ozone, generate reactive oxygen species (ROS) that disrupt tear film homeostasis and epithelial integrity, leading to “stinging, redness, and ocular surface inflammation” [26].

Serum 8-iso-PGF_2_α was inversely associated with diabetes (B = −0.35; 95% CI, 0.935–0.997; *p* = 0.033), a pattern that contrasts with the majority of reports linking elevated 8-iso-PGF_2_α to oxidative stress-related metabolic dysfunction. Prior investigations, including type 2 diabetic cohorts, have consistently demonstrated higher serum or urinary 8-iso-PGF_2_α levels in conjunction with increased fasting glucose, HbA1c, and visceral adiposity [27]. Additionally, a 2021 clinical study confirmed a strong positive correlation between plasma 8-iso-PGF_2_α and fasting blood glucose as well as intra-abdominal fat [28]. Even after controlling for key covariates—age, gender, smoking status, urinary 1-OHP, and CC16—our results revealed a significant negative association between 8-iso-PGF_2_α and diabetes status. This finding diverges from the conventional view that oxidative stress biomarkers are elevated in diabetes due to chronic hyperglycemia and insulin resistance. One plausible interpretation is that the observed inverse trend reflects an early-phase compensatory adaptation, where mild or subclinical metabolic stress elicits regulatory suppression of systemic lipid peroxidation. Such compensatory responses may involve increased antioxidant enzyme activity (e.g., superoxide dismutase, glutathione peroxidase) or altered membrane fatty acid composition that transiently suppresses 8-iso-PGF_2_α formation during prediabetic or early diabetic states [29]. Alternatively, the pattern could represent a phenotypic shift in oxidative response under environmental modulation. In populations exposed to intermittent PM_2.5_ pollution, like those living in Chiang Mai during biomass burning seasons, individuals with diabetes may develop metabolic adaptation mechanisms that blunt acute ROS-mediated lipid peroxidation, especially if concurrent inflammation or immune modulation dampens F2-isoprostane expression [30,31]. Finally, disease stage and medication use may affect oxidative biomarker levels. For example, metformin, a common antidiabetic drug, has been shown to reduce oxidative stress markers independent of glucose control [32]. To better contextualize our findings, Table 5 provides a comparative summary of biomarker responses reported in previous studies versus those observed in the present study.

While this study provides novel insights into the associations between PM_2.5_-related exposures, oxidative stress, and airway epithelial injury in a real-world setting, several limitations remain. First, the cross-sectional design inherently limits causal inference. Although key covariates such as age, gender, smoking status, personal protective behaviors (mask use and air purifier use), and co-exposure biomarkers (urinary 1-OHP, serum CC16, and 8-iso-PGF_2_α) were statistically controlled, temporality and directionality of associations cannot be definitively established. Second, although the study incorporated adjustments for personal protective measures, quantitative exposure assessment was based on ambient PM_2.5_ concentrations, which may not fully reflect individual-level inhalation doses. Factors such as indoor/outdoor mobility patterns and home ventilation conditions were not measured, possibly leading to exposure misclassification. Third, medication data were not collected, especially for participants with chronic conditions such as diabetes or hypertension. Since drugs like metformin, corticosteroids, or antihypertensives may influence oxidative stress and epithelial integrity, pharmacological confounding remains a possibility, particularly when interpreting biomarker variations across disease groups. Fourth, although the study employed validated symptom questionnaires and conducted subgroup comparisons, some response categories (e.g., “strongly agree”) had small sample sizes, which may limit statistical power and introduce instability in mean estimates. Fifth, this study focused on three key biomarkers—urinary 1-OHP, serum CC16, and 8-iso-PGF_2_α—representing exposure, epithelial injury, and oxidative stress, respectively. Other important biomarkers, such as systemic inflammation markers (e.g., CRP, IL-6), lung function parameters, or direct PAH adducts (e.g., BPDE-DNA adducts, malondialdehyde), were not measured. This limited biomarker panel may constrain the comprehensiveness of health effect evaluation, and future studies incorporating a broader biomarker spectrum are warranted. Finally, this investigation was conducted in a single urban region (Chiang Mai) during a seasonal haze episode, which, while relevant for studying biomass-related exposures, may limit the generalizability of findings to populations under different environmental or climatic conditions.

## 5. Conclusions

This study demonstrates that short-term exposure to elevated PM_2.5_ in Chiang Mai is associated with increased oxidative stress and early signs of airway epithelial injury. Elevated CC16 levels in smokers and symptomatic individuals may reflect acute epithelial responses, while higher 8-iso-PGF_2_α levels among those with pneumonia and eye irritation indicate enhanced lipid peroxidation. Notably, both CC16 and 8-iso-PGF_2_α showed significant associations with diabetes, with CC16 being positively related and 8-iso-PGF_2_α inversely related. Overall, CC16 and 8-iso-PGF_2_α appear to be useful biomarkers for assessing air pollution-related health risks, warranting further longitudinal investigation.

## Figures and Tables

**Table 1:** Baseline Characteristics and Biomarker Levels of the Study Population (N = 127).

Variables	Total (N = 127)
Age (years, mean ± SD)	59.7 ± 7.9
Sex: Male (*n*, %)	94 (74.0%)
Sex: Female (*n*, %)	33 (26.0%)
Smoking history: Yes (*n*, %)	20 (15.7%)
Body Mass Index (mean ± SD)	24.28 ± 8.14
Urinary 1-OHP (μmol/mol Cre)	0.88 ± 1.26
Serum CC16 (ng/mL, mean ± SD)	101 ± 48
Serum 8-iso-PGF2α (ng/mL, mean ± SD)	52.5 ± 20.6

Data are presented as means with standard deviations for continuous variables and as frequencies with percentages for categorical variables. Variables include age, sex, smoking history, BMI, and internal exposure biomarkers (urinary 1-OHP, serum CC16, and 8-iso-PGF2α).

**Table 2:** Comparison of Biomarker Levels by Demographic Characteristics.

Variables	CC16 (ng/mL)		8-iso-PGF2α Mean ± SD (ng/mL)		1-OHP Mean ± SD	
	Mean ± SD	*p* Value	Mean ± SD	*p* Value	Mean ± SD	*p* Value
Sex ^a^						
Male (74%)	100.1 ± 39.6	*p* > 0.05	51.3 ± 14.1	*p* > 0.05	0.87 ± 1.15	*p* > 0.05
Female (26%)	107.8 ± 67.2		56.0 ± 32.9		0.96 ± 1.39	
Age ^a^						
Age ≥ 60 (56.8%)	101.1 ± 39.8	*p* > 0.05	52.5 ± 20.5	*p* > 0.05	0.88 ± 1.26	*p* > 0.05
45 < Age < 60 (39%)	101.3 ± 60.4		51.3 ± 14.3		0.98 ± 1.27	
Age ≤ 45 (4.2%)	102.5 ± 22.5		45.6 ± 12.3		1.61 ± 2.31	
BMI ^a^						
BMI > 25 (31.4%)	92.9 ± 40.6	*p* > 0.05	47.9 ± 12.5	*p* > 0.05	0.72 ± 0.81	*p* > 0.05
23 < BMI < 25 (28%)	108.8 ± 67.5		54.7 ± 32.2		0.95 ± 1.63	
BMI < 23 (50.6%)	102.5 ± 36.1		54.3 ± 13.9		0.92 ± 1.27	
Smoke ^a^						
No	96.3 ± 39.9	*p* < 0.05 *	50.4 ± 13.8	*p* > 0.05	0.82 ± 1.18	*p* > 0.05
Yes	130.0 ± 65.3		62.7 ± 34.1		1.24 ± 1.74	
Alcohol ^a^						
No	101.5 ± 62.7	*p* > 0.05	50.8 ± 12.9	*p* > 0.05	0.91 ± 1.23	*p* > 0.05
Yes	101.7 ± 37.7		55.4 ± 29.7		0.81 ± 1.33	

^a^ Mann-Whitney U test. * *p* value < 0.05. Data are presented as mean ± standard deviation (SD); CC16 and 8-iso-PGF2α are serum biomarkers; 1-OHP is measured in urine (µmol/mol creatinine).

**Table 3:** Adjusted Associations Between Biomarker Levels and Self-Reported Symptoms.

Symptoms	Urinary 1-OHP ^a^ (μmol/mol Cre)		CC16 ^b^ (ng/mL)		8-iso-PGF2α ^c^ (ng/mL)	
	OR (95% CI)	*p*-Value	OR (95% CI)	*p*-Value	OR (95% CI)	*p*-Value
Eye irritation	0.740 (0.550–1.080)	0.131	0.989 (0.959–0.991)	0.002 **	1.049 (1.012–1.091)	0.01 **
Skin irritation	0.896 (0.647–1.255)	0.537	0.988 (0.976–1.000)	0.047 *	1.025 (0.996–1.055)	0.093
Respiratory irritation	0.783 (0.563–1.151)	0.234	0.990 (0.978–1.002)	0.096	1.031 (0.999–1.064)	0.054
Feeling short of breath	0.880 (0.636–1.236)	0.479	0.998 (0.987–1.010)	0.749	0.996 (0.969–1.024)	0.800
Dizzy	0.999 (0.700–1.426)	0.995	0.990 (0.978–1.003)	0.137	1.007 (0.976–1.039)	0.647
Fainting	0.777 (0.401–1.595)	0.526	0.990 (0.968–1.012)	0.372	1.009 (0.958–1.062)	0.741
Loss of consciousness	0.839 (0.475–1.527)	0.59	0.981 (0.963–0.999)	0.043 *	1.022 (0.979–1.067)	0.324
Pneumonia	0.841 (0.593–1.227)	0.391	0.980 (0.967–0.993)	0.002 **	1.039 (1.009–1.071)	0.012 *
Diabetes	1.157(0.834–1.640)	0.364	1.016 (1.002–1.030)	0.022 *	0.965 (0.935–0.997)	0.033 *

^a^ Adjusted for age, gender, smoking status, masks, air purifiers, F2α, and CC16. ^b^ Adjusted for age, gender, smoking status, masks, air purifiers, 1-OHP, and serum 8-iso-PGF2α. ^c^ Adjusted for age, gender, smoking status, masks, air purifiers, 1-OHP, and CC16. * *p* value < 0.05. ** *p* value < 0.01.

**Table 4:** Adjusted associations between self-reported respiratory symptom scores and biomarker levels (CC16, 8-iso-PGF_2_α, and 1-OHP) based on multivariate logistic regression.

Question	CC16		F2α		1-OHP	
	Mean ± SD	*p*	Mean ± SD	*p*	Mean ± SD	*p*
1. I have never had a cough						
0 (87.3%)	106.4 ± 48.1	0.003 *	53.4 ± 20.8	0.202	0.81 ± 1.14	0.159
1 (12.7%)	65.9 ± 30.3		45.7 ± 17.1		1.27 ± 1.85	
2. I have no phlegm in my lungs						
0 (94%)	103.8 ± 47.9	0.152	53.2 ± 20.3	0.305	0.87 ± 1.24	0.237
1 (6%)	60.4 ± 30.0		39.5 ± 23.0		0.79 ± 1.30	
3. I don’t feel tight in my chest						
0 (96.7%)	102.4 ± 48.1	0.948	53.2 ± 20.4	0.11	0.87 ± 1.26	0.925
1 (3.3%)	68.5 ± 39.4		32.4 ± 14.6		0.67 ± 0.63	
4. When I walk up a hill or up a flight of stairs, I can still breathe easily						
0 (85.6%)	106.6 ± 48.7	0.001 *	53.1 ± 21.1	0.449	0.84 ± 1.22	0.612
1 (14.4%)	69.9 ± 29.8		48.4 ± 16.1		0.98 ± 1.38	
5. I can do things at home without any restrictions						
0 (78.9%)	108.5 ± 47.7	0.001 *	53.2 ± 21.5	0.163	0.87 ± 1.25	0.833
1 (21.1%)	74.4 ± 35.23		49.7 ± 16.1		0.85 ± 1.24	
6. I have the confidence to go out, even though I have lung problems						
0 (85.6%)	106.2 ± 49.0	0.069	53.8 ± 21.0	0.546	0.90 ± 1.31	0.487
1 (14.4%)	72.2 ± 28.9		44.1 ± 14.7		0.61 ± 0.48	
7. I sleep very well						
0 (78.9%)	110.6 ± 47.7	0.001 *	54.3 ± 21.1	0.899	0.89 ± 1.27	0.841
1 (21.2%)	66.5 ± 31.2		45.4 ± 16.4		0.76 ± 1.16	
8. I feel very energetic						
0 (77.1%)	109.9 ± 48.1	0.001 *	53.6 ± 21.6	0.295	0.89 ± 1.28	0.943
1 (22.9%)	72.0 ± 35.4		48.4 ± 15.9		0.76 ± 1.13	

Note: Values are expressed as mean ± SD. *p*-values were obtained from multivariate binary logistic regression models adjusting for age, sex, and smoking status. Statistically significant *p*-values (*p* < 0.001) are marked with an asterisk (*). 0 = Disagree. 1 = Agree.

**Table 5:** Comparison of biomarker findings between this study and previous research.

Study (Author, Year)	Population/Location	Biomarkers Assessed	Main Findings	Comparison with Present Study
Current study (2025)	127 rural residents, Chiang Mai, Thailand	1-OHP, CC16, 8-iso-PGF_2_α	CC16 ↑ in smokers; CC16 ↓ in symptomatic groups (eye irritation, pneumonia, lack of energy); 8-iso-PGF_2_α ↑ in pneumonia and eye irritation; 8-iso-PGF_2_α ↓ in diabetes	Demonstrates mixed biomarker responses under seasonal haze exposure
Rostami et al., 2021 [19]	Smokers, airway epithelial samples	CC16	Smoking → CC16 ↓ due to club cell depletion	Contrasts with our CC16 ↑ in smokers, possibly reflecting compensatory secretion under haze stress
Tang et al., 2024 [17]	Human exposure study	CC16	Cigarette smoke exposure → CC16 ↓	Confirms conventional expectation, opposite to our finding
Jung et al., 2023 [20]	Biomass smoke exposure model	CC16	Biomass smoke → CC16 ↑ (acute secretion under oxidative stress)	Supports our interpretation of elevated CC16 in haze-exposed smokers
Nauwelaerts et al., 2022 [21]	Controlled PM exposure, general population	CC16 (serum/urine)	Short-term PM_2.5_ exposure → rapid CC16 ↑	Consistent with our observation that CC16 responds rapidly to exposure and symptoms
Neumann et al., 2024 [22]	Miners, occupational diesel/fire gas exposure	CC16	Acute rises in serum CC16 after exposure	Parallels our CC16 elevation under haze, but we also observed depletion in symptomatic individuals
Laing, 2019 [23]	Longitudinal birth cohort	CC16 + lung function (FEF_25–75_)	Higher CC16 → better small airway function	Supports our finding of positive correlation between CC16 and respiratory effort (respiratory rate)
Mukhtar et al., 2016 [27]	Type 2 diabetes cohort	8-iso-PGF_2_α	Diabetes → 8-iso-PGF_2_α ↑	Opposite to our finding (↓ in diabetes)
Ma et al., 2021 [29]	Clinical cohort, China	8-iso-PGF_2_α	Positive correlation with fasting glucose and visceral fat	Contrasts with our negative association in diabetes
Lim & Kim, 2024; [24] Sabir et al., 2025 [25]	Traffic-related PM and controlled PM_2.5_ exposure	8-iso-PGF_2_α	PM_2.5_ exposure → oxidative stress ↑ (lipid peroxidation)	Supports our finding of elevated 8-iso-PGF_2_α in pneumonia and eye irritation
Elfsmark et al., 2018 [26]	Ocular effects of pollutants	Oxidative stress mechanisms	ROS → eye irritation and ocular inflammation	Mechanistic support for our observed link between 8-iso-PGF_2_α and eye irritation

Note: The arrow meaning; ↑ = indicates an increase; ↓ = indicates a decrease; and → = indicates a causal or directional relationship.

## Data Availability

Data supporting the findings of this study are available from the corresponding author upon reasonable request.

## Abbreviations

The following abbreviations are used in this manuscript:

PAHs	Polycyclic Aromatic Hydrocarbons
PM_2.5_	Particulate Matter with an aerodynamic diameter ≤ 2.5 µm
1-OHP	1-Hydroxypyrene
CC16	Club Cell Secretory Protein 16
8-iso-PGF_2_α	8-iso-Prostaglandin F_2_α
ELISA	Enzyme-Linked Immunosorbent Assay
HPLC	High-Performance Liquid Chromatography
BMI	Body Mass Index
NTAQHI	Northern Thailand Air Quality Health Index
